# Early Risk Stratification for Subsequent Small Airway Dysfunction in Hospitalized Children with *Mycoplasma Pneumoniae* Pneumonia: A Retrospective Cohort Study

**DOI:** 10.3390/children13050713

**Published:** 2026-05-21

**Authors:** Ruimeng Ma, Jingrong Song, Yu Fu, Rui Li, Tienan Feng, Zonglang Yu, Mengting Zhang, Shuping Jin, Xiaoying Zhang

**Affiliations:** 1 Shanghai Ninth People’s Hospital, Shanghai Jiao Tong University School of Medicine, No. 639, Zhi Zao Ju Road, Shanghai 200011, China; sakura666@sjtu.edu.cn (R.M.); songyang92006@163.com (J.S.); fuyu18856030890@163.com (Y.F.); 72300320004@shsmu.edu.cn (R.L.); yuzonglang@sjtu.edu.cn (Z.Y.); zmtaaaaa@163.com (M.Z.); 330483200108230066@sjtu.edu.cn (S.J.); 2 Institute of Clinical Medicine, Shanghai Jiao Tong University School of Medicine, No. 227 Chongqing South Road, Shanghai 200011, China; tienan_feng@126.com

**Keywords:** *Mycoplasma pneumoniae* pneumonia, small airway dysfunction, risk stratification, chest computed tomography, pulmonary function

## Abstract

**Highlights:**

**What are the main findings?**

- Small airway dysfunction was common in hospitalized children with *Mycoplasma pneumoniae* pneumonia and was identified in 44.2% of the study cohort.
- A model based on routinely available clinical and chest CT variables showed good performance for early risk stratification.

**What are the implications of the main findings?**

- Early bedside risk stratification may help identify children who are more likely to require pulmonary function testing after clinical stabilization to confirm small airway dysfunction.
- A practical model based on readily available clinical and imaging data may support more selective use of pulmonary function testing and improve allocation of clinical resources in hospitalized children with MPP.

**Abstract:**

**Background/Objectives**: Small airway dysfunction (SAD) may occur early in children with *Mycoplasma pneumoniae* pneumonia (MPP), but pulmonary function testing is often deferred until clinical stabilization and may be limited by poor cooperation. Early risk stratification may therefore help identify children who warrant further testing. We aimed to identify early predictors of subsequent spirometry-defined SAD and to develop an internally validated risk-stratification model in hospitalized children with MPP. **Methods**: In this single-center retrospective cohort study conducted between July 2022 and July 2024, 172 hospitalized children with confirmed MPP were included. Clinical characteristics, immune-inflammatory indices, and chest computed tomography (CT) findings were collected during early hospitalization. Pulmonary function testing was performed after clinical stabilization, and SAD was defined as at least two of forced expiratory flow at 25%, 50%, and 75% of forced vital capacity being <65% of predicted values. Multiple imputation, LASSO selection, and multivariable logistic regression were used for model development and bootstrap internal validation. **Results**: SAD was identified in 76/172 children (44.2%). Wheezing, CT evidence of small airway involvement, and higher soluble interleukin-2 receptor levels were more common in children with SAD; wheezing remained independently associated with SAD. A model based on routine clinical and chest CT variables showed good discrimination (AUC, 0.885; optimism-corrected AUC, 0.869). Adding interleukin-17 provided limited incremental value. **Conclusions**: SAD was common in hospitalized children with MPP. An internally validated model based on readily available clinical and chest CT variables may help prioritize children for pulmonary function testing after clinical stabilization, whereas interleukin-17 added limited predictive value. External validation is required before broader clinical application.

## 1. Introduction

*Mycoplasma pneumoniae* pneumonia (MPP) is a major cause of community-acquired pneumonia in school-aged children [1,2] and may lead to a broad spectrum of airway and pulmonary complications [3,4,5,6]. In addition to parenchymal inflammation, MPP may involve the peripheral airways and cause airflow limitation that is not readily recognized during routine clinical evaluation [7,8,9]. Small airway dysfunction (SAD) has attracted increasing attention because it may occur early in the disease course and may be associated with persistent respiratory symptoms and subsequent airway morbidity [10,11]. Early identification of children at increased risk of subsequent SAD may therefore help prioritize targeted pulmonary function testing after clinical stabilization and support follow-up planning.

Pulmonary function testing remains the standard method for assessing airflow limitation and identifying spirometric abnormalities suggestive of SAD [12]. In hospitalized children with acute pneumonia, however, testing is often constrained by age, poor cooperation, and the timing of assessment, and is commonly deferred until the child’s condition has improved sufficiently to allow reliable performance [5]. As a result, early clinical decision-making more often relies on routinely available bedside manifestations, laboratory findings, and imaging features. Previous studies in children with MPP have identified factors associated with severe or refractory disease, including wheezing, inflammatory markers, and radiographic abnormalities [13,14,15,16], but have mainly focused on overall disease severity rather than early identification of children who may subsequently have SAD and warrant confirmatory pulmonary function testing.

Chest computed tomography (CT) may provide additional information on airway and parenchymal abnormalities in children with MPP, including bronchial wall thickening, mosaic attenuation, and other findings suggestive of peripheral airway involvement [17,18]. Immune-inflammatory markers, such as cytokines and lymphocyte subsets, may further reflect host responses related to airway injury [19,20,21]. However, no previous study has integrated clinical features, chest CT findings, and immune-inflammatory markers into an early risk-stratification model for subsequent spirometry-defined SAD in hospitalized children with MPP. Therefore, this study aimed to identify early factors associated with subsequent spirometry-defined SAD and to develop a risk-stratification model based on routinely available in-hospital data to help guide selective pulmonary function testing in this population.

## 2. Materials and Methods

### 2.1. Study Design

We retrospectively reviewed the medical records of children aged 6–17 years who were hospitalized with confirmed MPP at Shanghai Ninth People’s Hospital, Shanghai Jiao Tong University School of Medicine, between July 2022 and July 2024. Candidate predictors were derived from routinely collected clinical characteristics, laboratory and immune-inflammatory indices, and chest computed tomography (CT) findings obtained during early hospitalization, whereas the study outcome was subsequent SAD determined by pulmonary function testing after clinical stabilization. The lower age limit of 6 years was chosen to ensure acceptable spirometric performance for outcome assessment. The study was conducted in accordance with the Declaration of Helsinki and was approved by the Medical Ethics Committee of Shanghai Ninth People’s Hospital, Shanghai Jiao Tong University School of Medicine (approval no. SH9H-2024-T331-1, 11 September 2024). Given the retrospective design and use of existing clinical data, the requirement for study-specific informed consent was waived by the ethics committee. Guardians are routinely informed at admission that de-identified clinical data may be used for ethics-approved retrospective studies and may decline such use. Children whose guardians declined authorization were not included in the present study.

### 2.2. Participants and Eligibility Criteria

MPP was diagnosed in accordance with the Guideline for Diagnosis and Treatment of *Mycoplasma pneumoniae* Pneumonia in Children (2023 edition) [22]. Children were eligible if they met all of the following criteria: (1) age 6–17 years; (2) hospitalization during the study period; (3) presentation within 2 weeks of disease onset with acute respiratory symptoms, including cough, fever, or other respiratory manifestations; (4) microbiological evidence of *M. pneumoniae* infection, defined as at least one positive polymerase chain reaction result from a biological sample, a single serum *M. pneumoniae* antibody titer ≥1:160 by particle agglutination assay, or a ≥4-fold rise in paired serum antibody titers during the course of illness; and (5) radiologically confirmed pneumonia on chest radiography or CT, manifested as patchy infiltrates, segmental or lobar consolidation. Segmental or lobar atelectasis and pleural effusion were recorded as accompanying imaging findings rather than standalone diagnostic criteria.

Children were excluded if they had: (1) pre-existing chronic respiratory diseases that could substantially affect small airway function or spirometric interpretation, including asthma, bronchiectasis, congenital airway or lung developmental abnormalities, interstitial lung disease, chronic obstructive pulmonary disease, or pulmonary embolism; (2) systemic diseases that might influence pulmonary function measurements, such as malignancy, heart failure, or rheumatic disease; (3) incomplete clinical data; (4) unavailable or unacceptable pulmonary function testing for assessment of the study outcome; or (5) declined authorization for retrospective research use of de-identified clinical data.

### 2.3. Data Collection

Candidate predictors were collected during the early hospitalization period from the electronic medical record system, routine laboratory testing, immune-inflammatory assays, and chest computed tomography (CT). Demographic and clinical variables included age, sex, height, weight, presenting symptoms and respiratory signs, fever course (days of fever before blood sampling and total duration of fever), treatment exposure before blood sampling (days of corticosteroid therapy and prior macrolide use), and past medical history, including previous wheezing, prior pneumonia, food allergy, eczema, and allergic rhinitis.

Laboratory and immune-inflammatory data were obtained from the first blood test performed within 24 h of admission. Peripheral venous blood samples were collected within 24 h after hospitalization. Recorded variables included white blood cell count, neutrophil count and percentage, lymphocyte count and percentage, eosinophil count and percentage, C-reactive protein, lactate dehydrogenase, erythrocyte sedimentation rate, ferritin, cytokines, and immune cell subsets. Immune-inflammatory markers were extracted retrospectively from tests that had already been ordered during routine inpatient care; no additional assays were performed specifically for this study.

In the present analytic cohort, chest CT had been performed within 24 h of admission as part of routine clinical care and was available for structured review. Chest CT findings were assessed using a structured scoring approach adapted from previously reported pediatric pneumonia CT scoring methods [23,24,25]. All imaging studies were independently reviewed by two radiologists who were blinded to the outcome classification, and disagreements were resolved by a third radiologist. The composite chest CT score comprised three components: a pulmonary parenchymal involvement score, an extrapulmonary involvement score, and a small airway involvement score. In addition, specific airway-related imaging signs, including bronchial wall thickening and mosaic attenuation, were recorded for subsequent analyses and model development.

For pulmonary parenchymal involvement, a “highest severity predominates” principle was applied, such that different parenchymal abnormalities were not scored cumulatively. Patchy or linear opacities and small patchy opacities were assigned 1 point, whereas large consolidation or atelectasis was assigned 3 points. If more than one parenchymal abnormality was present, only the most severe lesion was scored. The extrapulmonary involvement score was calculated cumulatively according to the presence of pleural effusion (1 point) and pleural involvement (1 point; range, 0–2). The small airway involvement score was also cumulative and assigned according to the presence of mosaic attenuation (1 point) and bronchial wall thickening (1 point; range, 0–2).

### 2.4. Pulmonary Function Testing and Outcome Definition

All enrolled children received standard treatment after admission in accordance with the Guideline for Diagnosis and Treatment of *Mycoplasma pneumoniae* Pneumonia in Children (2023 edition) [22]. Pulmonary function testing was performed after clinical stabilization using the Master-Screen Pediatric pulmonary function system (Jaeger, Germany) according to pediatric spirometry procedures recommended by the American Thoracic Society/European Respiratory Society [12,26]. The recorded parameters included forced expiratory volume in 1 s, forced vital capacity, forced expiratory volume in 1 s/forced vital capacity ratio, forced expiratory flow at 25%, 50%, and 75% of forced vital capacity (FEF25, FEF50, and FEF75, respectively), and maximal mid-expiratory flow. Spirometric indices were expressed as percentages of predicted values generated by the pulmonary function system using pediatric reference equations routinely applied in our laboratory, taking age, sex, and height into account.

The study outcome was subsequent spirometry-defined small airway dysfunction (SAD). In this study, SAD was prespecified as an operational definition based on the prior small-airway literature and defined as at least two of FEF25, FEF50, and FEF75 being <65% of predicted values. These mid-expiratory flow indices were selected because they are commonly used as spirometric markers of small-airway flow limitation [4,9]; however, they are also sensitive to expiratory effort and lung volume. Requiring at least two abnormal indices was intended to improve robustness and reduce misclassification related to variability in a single parameter. We acknowledge that z-score- or lower-limit-of-normal-based approaches are also reasonable for physiologic interpretation, but no universally accepted z-score-based definition of spirometry-defined SAD currently exists for hospitalized children with MPP.

### 2.5. Bronchoscopy and Bronchoalveolar Lavage

Flexible bronchoscopy with bronchoalveolar lavage was not mandated by the study protocol and was performed selectively according to clinical need for diagnostic and/or therapeutic purposes, particularly in children with persistent segmental/lobar atelectasis, large or multilobar consolidation, suspected endobronchial mucus plugging/plastic bronchitis, or poor clinical/radiologic response to standard treatment. None of the enrolled children required PICU admission or mechanical ventilation, and no child was intubated solely for bronchoalveolar lavage.

### 2.6. Statistical Analysis

All statistical analyses were performed using R software (version 4.5.2). Continuous variables are presented as median (interquartile range, IQR), and categorical variables as counts and percentages. Between-group comparisons were performed using the Wilcoxon rank-sum test for continuous variables and Fisher’s exact test for categorical variables. Missing data in candidate predictors were handled using multiple imputation by chained equations, generating 20 imputed datasets, and regression estimates were pooled according to Rubin’s rules. Continuous laboratory and immune-inflammatory variables with skewed distributions were log1p-transformed before regression analyses.

Candidate predictors available during early hospitalization were entered into least absolute shrinkage and selection operator (LASSO)-penalized logistic regression for variable selection, with the penalty parameter determined by cross-validation. Variables retained after LASSO selection, together with sex as a prespecified covariate, were entered into multivariable logistic regression models. A base model (BASE) was constructed using routinely available demographic, clinical, and chest CT variables, and an extended model (EXT) was developed by additionally including interleukin-17 (IL-17). Nomograms were generated on the basis of the final regression coefficients. Among the assayed immune-inflammatory markers, log1p-transformed IL-17 was the only cytokine retained with a non-zero coefficient after LASSO selection; it was therefore included in the extended model to assess the incremental value of biologically plausible immune information beyond routinely available clinical variables.

Model performance was assessed by discrimination, calibration, and clinical utility. Discrimination was evaluated using the area under the receiver operating characteristic curve (AUC), calibration using calibration plots, intercepts, and slopes, and clinical utility using decision curve analysis. Internal validation was performed using 1000 bootstrap resamples to estimate optimism-corrected model performance. The model was developed for risk prediction rather than etiologic inference. Accordingly, predictor retention was guided by penalized selection, clinical plausibility, and contribution to overall model performance, and individual regression coefficients were not intended to support causal interpretation.

All statistical tests were two-sided, and *p* < 0.05 was considered statistically significant.

## 3. Results

### 3.1. Study Population and Outcome Classification

**Figure 1:**
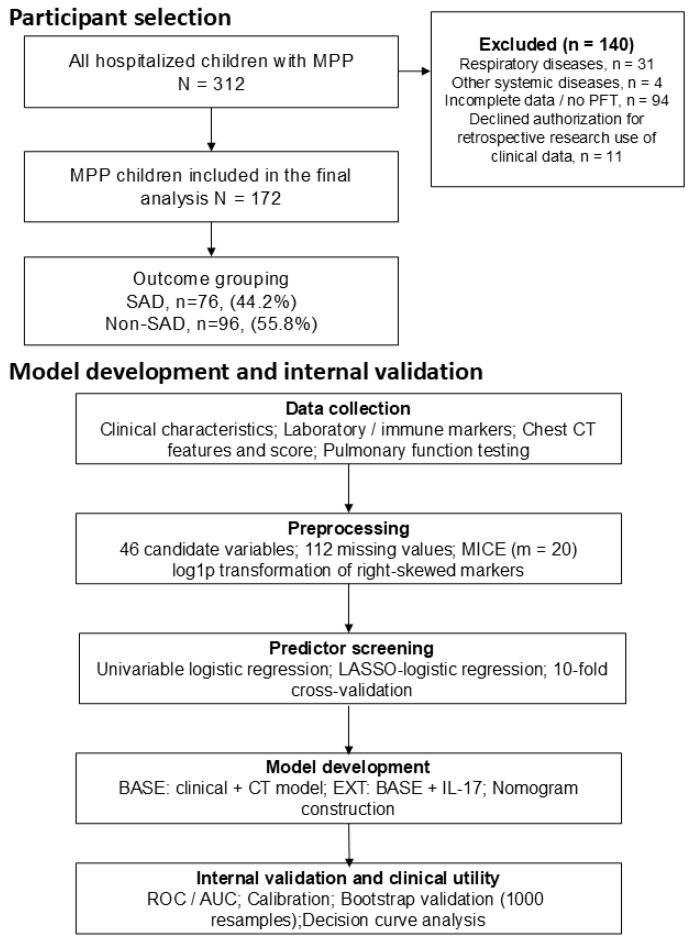
Study flowchart showing participant selection, outcome classification, and model development with internal validation.

A total of 312 hospitalized children with *Mycoplasma pneumoniae* pneumonia (MPP) were screened for eligibility. After exclusion of children with pre-existing respiratory disease (n = 31), coexisting systemic disease (n = 4), incomplete clinical data or unavailable pulmonary function testing (n = 94), and declined authorization for retrospective research use of de-identified clinical data (n = 11), 172 children were included in the final analysis. Among these, 76 children (44.2%) were classified as having subsequent spirometry-defined small airway dysfunction (SAD), whereas 96 (55.8%) were classified as non-SAD. Accordingly, the observed frequency of SAD should be interpreted as applying to children with available and acceptable spirometry after clinical stabilization, rather than to all hospitalized children with MPP. The participant selection process is shown in Figure 1.

### 3.2. Baseline Characteristics

Baseline characteristics are summarized in Table 1. Compared with the non-SAD group, children with SAD were younger and had more pronounced radiologic involvement, as reflected by a higher total chest CT score. Wheezing and moist rales were also more common in the SAD group. In addition, children with SAD were more likely to have wheezing within the previous year, previous pneumonia, and a history of eczema. Imaging findings suggestive of airway-predominant disease, including bronchial wall thickening, mosaic attenuation, and higher CT small airway involvement scores, were more frequent in the SAD group. Among the immune-inflammatory markers, soluble interleukin-2 receptor (sIL-2R) levels were higher in children with SAD, whereas most other laboratory and immune variables did not differ significantly between groups. Detailed baseline data are provided in Table 1 and Supplementary Table S1.

**Figure 2:**
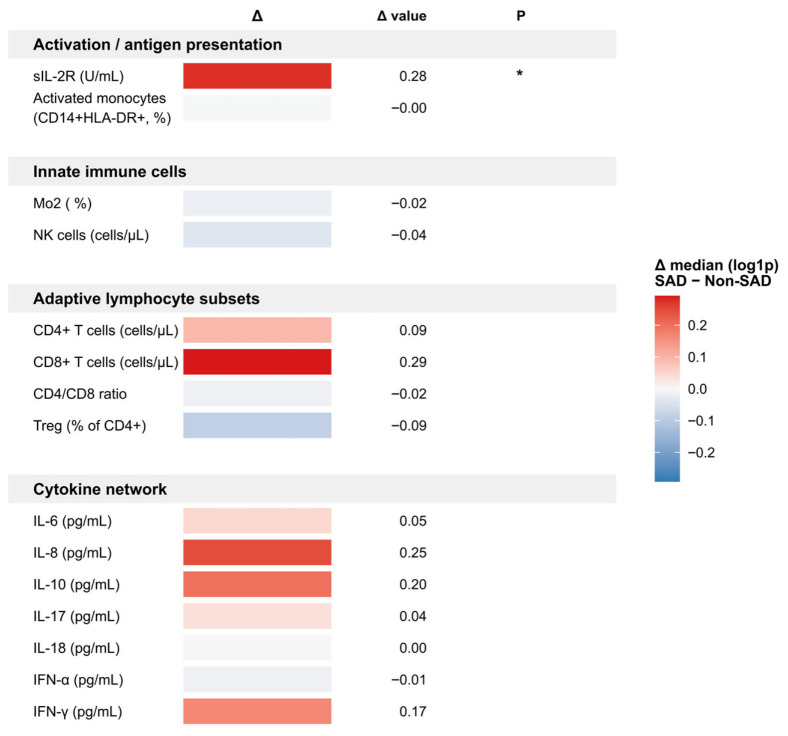
Immune landscape associated with small airway dysfunction in children with *Mycoplasma pneumoniae* pneumonia. * The heatmap displays between-group differences in log1p-transformed median values, calculated as Δ = median[log(x + 1)]_SAD_ − median[log(x + 1)]_Non−SAD_. Positive Δ values (red) indicate higher levels in the SAD group, whereas negative Δ values (blue) indicate lower levels in the SAD group. The corresponding Δ values are shown numerically, and statistical significance was assessed using two-sided Wilcoxon tests; * indicates *p* < 0.05.

To further characterize immune features according to outcome status, immune markers were grouped into functional modules and visualized as an immune landscape heatmap (Figure 2). Within this analysis, sIL-2R showed a significant upward shift in the SAD group, whereas most other immune cell subsets and cytokines showed only modest directional differences without statistical significance.

### 3.3. Missing Data

A total of 46 candidate variables were included in the analysis, with 112 missing observations overall. Variable-specific missingness ranged from 0.6% to 7.6%. Given the low overall level of missingness, multiple imputation by chained equations was used, and subsequent regression modeling and performance evaluation were conducted in the imputed datasets. Variable-specific missingness rates are shown in Supplementary Table S2.

### 3.4. Candidate Predictor Assessment and LASSO Selection

**Figure 3:**
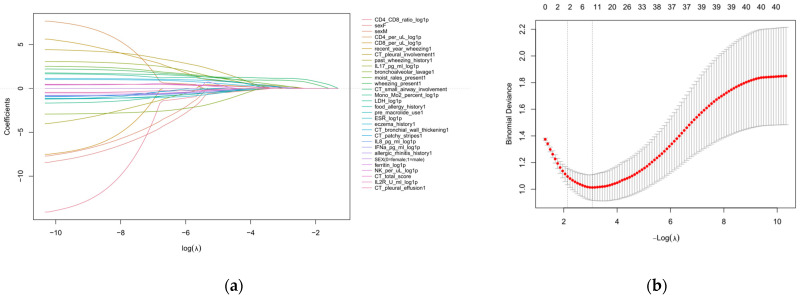
LASSO coefficient profiles and cross-validation for predictor selection in the SAD prediction model: (**a**) Coefficient trajectories of candidate predictors across different values of log(λ); (**b**) Ten-fold cross-validation curve showing mean binomial deviance across values of −log(λ). Red points/curve indicate the mean cross-validated binomial deviance, grey error bars indicate ±1 standard error, and vertical dashed lines indicate λ_min_ and λ_1se_.

Exploratory univariable analyses showed that wheezing, moist rales, imaging evidence of small airway involvement, and higher total chest CT scores were associated with SAD; full results are provided in Supplementary Table S3. LASSO-penalized logistic regression was then applied for predictor shrinkage and selection. Using λ_min_ as the selection criterion, the variables retained with non-zero coefficients were age, sex, wheezing, moist rales, wheezing within the previous year, eczema history, bronchial wall thickening on CT, CT small airway involvement score, chest CT total score, and log1p-transformed IL-17 (Figure 3). Considering clinical practicality, these results were used to construct a base model (BASE) using routinely available clinical and chest CT variables and an extended model (EXT) by additionally incorporating IL-17.

### 3.5. Multivariable Model Development and Nomogram Construction

**Figure 4:**
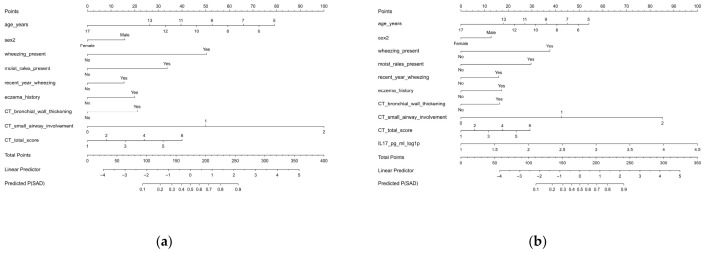
Nomograms for predicting small airway dysfunction in hospitalized children with *Mycoplasma pneumoniae* pneumonia. (**a**) Nomogram of the BASE model, which includes age, sex, wheezing, moist rales, wheezing within the previous year, history of eczema, bronchial wall thickening on chest computed tomography (CT), CT small airway involvement score, and chest CT total score. (**b**) Nomogram of the EXT model, which includes all predictors in the BASE model plus log1p-transformed interleukin-17 (IL-17). For each predictor, the corresponding number of points is assigned according to its value on the nomogram scale. The total points are obtained by summing the points for all predictors and are then projected to estimate the individual probability of small airway dysfunction (SAD). Higher total points indicate a higher predicted risk of SAD.

Multivariable logistic regression models were developed in the multiply imputed dataset on the basis of the predictors retained after LASSO selection, with sex included as a prespecified covariate. In the BASE model, wheezing remained independently associated with SAD after adjustment for the other variables (OR, 3.70; 95% CI, 1.25–10.89; *p* = 0.018). The remaining predictors were retained in the final model on the basis of LASSO stability, clinical relevance, and contribution to overall model performance. To assess the incremental value of immune-inflammatory information beyond routinely available variables, IL-17 was added to generate the EXT model. In the EXT model, wheezing also remained independently associated with SAD (OR, 3.37; 95% CI, 1.14–9.96; *p* = 0.028), whereas IL-17 showed a positive but non-significant association (OR, 2.47; 95% CI, 0.79–7.65; *p* = 0.117). The full multivariable results are shown in Table 2. Nomograms based on the final coefficients of the BASE and EXT models are presented in Figure 4.

### 3.6. Discrimination, Internal Validation, and Calibration

**Figure 5:**
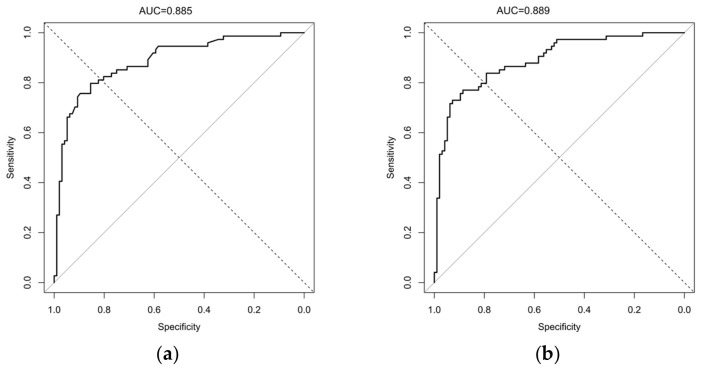
Receiver operating characteristic curves of the BASE and EXT models for predicting small airway dysfunction in hospitalized children with *Mycoplasma pneumoniae* pneumonia. (**a**) Receiver operating characteristic curve of the BASE model. (**b**) Receiver operating characteristic curve of the EXT model. The solid ROC curves show the discriminative performance of each model across different classification thresholds, whereas the diagonal dashed reference line represents no discriminative ability (AUC = 0.5). The area under the curve (AUC) was 0.885 for the BASE model and 0.889 for the EXT model, indicating good discriminative performance for both models.

**Figure 6:**
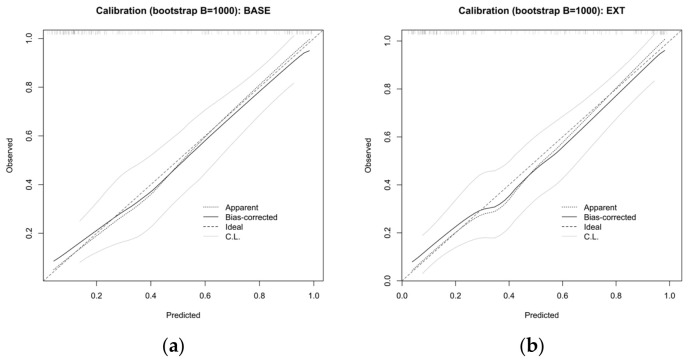
Bootstrap-corrected calibration curves of the BASE and EXT models for predicting small airway dysfunction in hospitalized children with *Mycoplasma pneumoniae* pneumonia. (**a**) Bootstrap-corrected calibration curve of the BASE model. (**b**) Bootstrap-corrected calibration curve of the EXT model. Calibration was assessed using 1000 bootstrap resamples.

Both models showed good discrimination. The AUC was 0.885 for the BASE model and 0.889 for the EXT model (Figure 5). After bootstrap internal validation with 1000 resamples, the optimism-corrected AUCs remained similar at 0.869 for the BASE model and 0.872 for the EXT model, indicating limited incremental discrimination from adding IL-17. Calibration was also good for both models. Bootstrap-corrected calibration curves showed close agreement between predicted and observed risk, and both models had calibration intercepts close to 0 and slopes close to 1 (Figure 6).

### 3.7. Decision Curve Analysis

**Figure 7:**
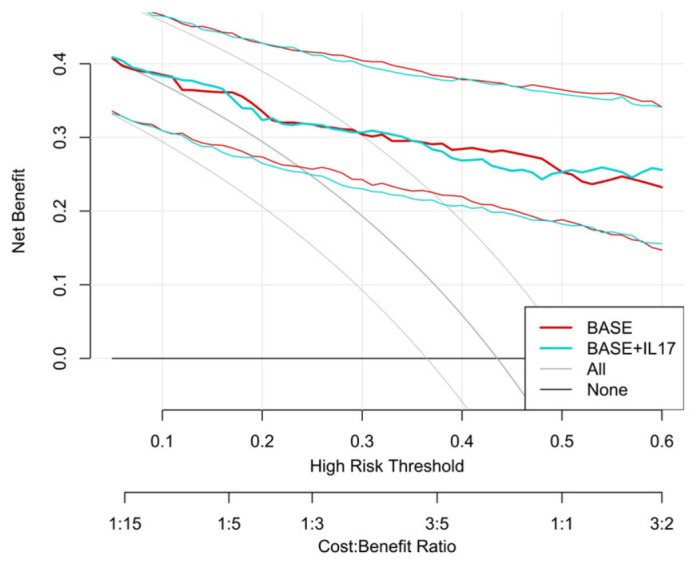
Decision curve analysis of the BASE and EXT models for predicting small airway dysfunction in hospitalized children with *Mycoplasma pneumoniae* pneumonia.

Decision curve analysis showed that, across a threshold probability range of approximately 0.10–0.60, both the BASE and EXT models provided greater net benefit than the treat-all and treat-none strategies (Figure 7). However, the net benefit curves of the BASE and EXT models were largely overlapping, and the incremental net benefit of adding IL-17 was minimal overall (Supplementary Figure S2). These findings suggest that a model based on routinely available clinical and chest CT variables may already provide most of the useful information for early in-hospital risk stratification of children who may warrant further pulmonary function testing after clinical stabilization.

## 4. Discussion

In this retrospective cohort of hospitalized children with *Mycoplasma pneumoniae* pneumonia (MPP), subsequent spirometry-defined small airway dysfunction (SAD) was common. A risk-stratification model based on routinely available clinical and chest computed tomography (CT) variables obtained early during hospitalization showed good discrimination and calibration in this cohort. By contrast, adding interleukin-17 (IL-17) provided only limited incremental improvement in model performance and decision-curve net benefit. Taken together, these findings suggest that, in hospitalized children with MPP, routinely available clinical and imaging information may provide a practical basis for early in-hospital risk stratification before confirmatory pulmonary function testing after clinical stabilization.

Previous prediction models in children with MPP have mainly focused on severe or refractory disease and on complications such as plastic bronchitis, bronchial mucus plugs, or bronchiolitis obliterans [13,14,15,27], whereas the present study focused on SAD as a functional airway outcome. This distinction may be clinically relevant because SAD may occur even when the overall clinical picture appears to be improving, and functional airway impairment may not be fully captured by symptom relief or radiologic resolution alone [10,28]. In this context, our findings support the concept that early bedside information may help identify children who are more likely to have subsequent spirometry-defined SAD and who may therefore warrant pulmonary function testing and closer follow-up after stabilization [4]. The model should therefore be viewed as a risk-stratification tool to support selective assessment, rather than as a substitute for pulmonary function-based confirmation.

The spirometry-defined SAD outcome used in this study should be interpreted as a pragmatic, literature-based operational definition rather than a definitive physiologic gold standard [4,12,26]. Mid-expiratory flow indices may provide clinically useful information on peripheral airway involvement, particularly when conventional spirometric indices remain near normal. However, they are also effort- and volume-sensitive and should not be overinterpreted in isolation [12,26]. In this study, requiring at least two abnormal indices was intended to improve robustness and reduce misclassification related to variability in a single parameter. We also acknowledge that z-score- or LLN-based approaches may offer a more standardized framework for physiologic interpretation and deserve further evaluation in future pediatric MPP studies.

From a clinical perspective, this model may help identify children who are more likely to have subsequent SAD and therefore may benefit from prioritized spirometry after clinical stabilization, closer outpatient follow-up, and earlier pediatric pulmonology assessment if respiratory symptoms persist. Wheezing and moist rales may indicate more prominent acute airway involvement, including airway narrowing, secretion retention, and peripheral airway inflammation during the acute phase of infection [2,8]. By contrast, a history of wheezing and eczema may suggest an underlying susceptibility to airway hyperresponsiveness or an atopic background [29], which could make some children more vulnerable to small airway impairment after *M. pneumoniae* infection. These interpretations remain cautious, but they are broadly consistent with previous reports linking airway symptoms, atopic characteristics, and post-infectious small-airway sequelae in pediatric respiratory disease [15,16,30,31,32]. Accordingly, even when individual predictors did not all reach conventional statistical significance in the multivariable model, they may still contribute useful clinical information within an integrated early risk-stratification framework.

Imaging variables also contributed meaningfully to the present model. Bronchial wall thickening, CT-defined small airway involvement, and the total chest CT score were retained in the final model and may reflect both the extent of acute pulmonary involvement and an airway-predominant pattern of disease. Previous studies have suggested that children with MPP who show prominent airway abnormalities on imaging may be at increased risk of persistent airway sequelae, including post-infectious bronchiolitis obliterans [17,18,30]. In this context, our findings support the view that CT features suggestive of small airway involvement may serve as structural correlates of subsequent spirometry-defined SAD. From a practical perspective, these imaging findings may be helpful for early in-hospital risk stratification when pulmonary function testing is not yet feasible [3,4,7,9].

To further explore the biologic context of SAD, we examined immune markers and constructed an immune landscape. Overall, the between-group pattern appeared to suggest relatively greater immune activation together with less prominent regulatory signals in children with SAD, although most immune differences were modest and should be interpreted cautiously. Among these markers, soluble interleukin-2 receptor (sIL-2R) was higher in the SAD group, which may indicate stronger T-cell activation in children who subsequently show spirometric evidence of small airway impairment [19,33,34]. Upward trends in cytokines such as IL-8, IL-10, and IFN-γ may also be compatible with a more activated inflammatory state [21,35,36]; however, these findings were exploratory and do not establish a specific mechanistic pathway. Differences in innate immune and monocyte-related markers were relatively limited, which may reflect the constraints of single-time-point peripheral blood sampling rather than the absence of biologic relevance.

The IL-17/Th17 pathway may be of potential interest in this setting, as previous studies have linked serum IL-17 to disease activity in pediatric MPP [37,38,39]. In our cohort, IL-17 showed a modest upward shift in children with SAD, and the immune landscape also suggested relatively lower regulatory T-cell proportions [40]. However, when IL-17 was added to the BASE model, the gain in discrimination and clinical net benefit was limited. Several factors may account for this finding. First, routinely available clinical and CT variables may already capture much of the relevant inflammatory and airway burden. Second, serum IL-17 was measured at a single time point and may have been influenced by sampling timing, prior treatment exposure, and interindividual immune heterogeneity. Third, systemic cytokine levels may not fully reflect the local airway immune microenvironment [19,34]. This apparent discrepancy between biologic plausibility and limited predictive gain may reflect the fact that serum IL-17 is dynamic, may vary with sampling time and prior treatment, and may not fully capture the local airway inflammatory microenvironment relevant to later spirometric dysfunction. Taken together, these findings suggest that although IL-17 may have biologic relevance, it did not provide sufficient incremental value to support routine inclusion in an early bedside risk-stratification model in this cohort. Accordingly, the present data do not support routine serum cytokine testing, including IL-17, for bedside risk stratification of subsequent SAD in hospitalized children with MPP.

From a clinical perspective, the BASE model is composed of routinely available bedside information, including medical history, physical signs, and chest CT features, and showed stable performance after internal validation. It may therefore help support early in-hospital risk stratification in children with MPP and may assist clinicians in prioritizing pulmonary function testing after clinical stabilization, particularly when universal testing is impractical. The model should be regarded as a tool to support selective assessment rather than as a substitute for spirometry-based confirmation.

Given the modest number of outcome events relative to the number of predictor parameters, the present model should be interpreted primarily as a pragmatic prediction tool rather than an explanatory model. Although penalization and bootstrap validation were used to reduce overfitting, coefficient estimates remain subject to uncertainty. Therefore, the individual odds ratios, particularly for predictors retained for overall model performance rather than conventional statistical significance, should not be overinterpreted as precise effect estimates.

Several limitations should be acknowledged. First, this was a single-center retrospective study and may therefore be affected by residual bias and limited generalizability. Second, because pulmonary function testing was not available or acceptable in all screened children, selection bias cannot be excluded. In pediatric practice, spirometry feasibility is influenced by age and cooperation [41]. Therefore, the observed frequency of SAD in this study should not be interpreted as the burden in all hospitalized children with MPP, and the model may be less directly applicable to children in whom reliable spirometry cannot be obtained. Third, as none of the enrolled children required PICU admission or mechanical ventilation, the cohort mainly represented non-critically ill hospitalized children; accordingly, the applicability of the model to critically ill MPP populations remains uncertain. Fourth, the model has not yet undergone external validation. Finally, the inclusion of chest CT variables may limit applicability in settings where CT is not routinely performed. Therefore, the findings should be interpreted cautiously. Further multicenter prospective studies are warranted to externally validate and refine the model and to clarify its role in guiding pulmonary function assessment and follow-up in broader pediatric MPP populations.

We agree that future studies should explore whether less invasive bedside modalities, particularly lung ultrasound, can provide structural surrogates of airway-predominant involvement and be incorporated into simplified risk-stratification models for children.

## 5. Conclusions

In hospitalized children with *Mycoplasma pneumoniae* pneumonia, subsequent spirometry-defined small airway dysfunction was common. A model based on routinely available clinical and chest CT variables may help support early in-hospital risk stratification and prioritize pulmonary function testing after clinical stabilization. The model should be interpreted as a tool for selective assessment rather than diagnostic replacement, and external validation is required before broader use.

## Figures and Tables

**Table 1:** Baseline characteristics of hospitalized children with *Mycoplasma pneumoniae* pneumonia (MPP) stratified by small airway dysfunction (SAD).

Characteristic	Non-SAD (n = 96)	SAD (n = 76)	*p*-Value
Demographics and clinical course			
Age, years	7.00 (6.00, 9.00)	6.00 (6.00, 8.00)	0.031
Male sex, n (%)	41 (42.7%)	31 (40.8%)	0.515
Wheezing at admission, n (%)	10 (10.4%)	40 (52.6%)	<0.001
Moist rales at admission, n (%)	57 (59.4%)	66 (86.8%)	<0.001
Bronchoalveolar lavage performed, n (%)	14 (14.6%)	17 (22.4%)	0.232
Pre-hospital macrolide use, n (%)	91 (94.8%)	66 (86.8%)	0.101
Days before systemic corticosteroid initiation, days	3.00 (1.00, 4.00)	3.00 (1.00, 5.00)	0.208
Fever duration before sampling, days	5.00 (4.00, 7.00)	6.00 (3.00, 7.25)	0.957
Past history and atopic background			
Wheezing in the past year, n (%)	15 (15.6%)	35 (46.1%)	<0.001
Previous pneumonia, n (%)	40 (41.7%)	51 (67.1%)	0.001
History of eczema, n (%)	33 (34.4%)	42 (55.3%)	0.008
Chest CT characteristics			
Chest CT total score, points	2.00 (1.00, 3.00)	4.00 (3.00, 5.00)	<0.001
Large consolidation or atelectasis on CT, n (%)	43 (44.8%)	53 (69.7%)	0.001
Bronchial wall thickening on CT, n (%)	8 (8.3%)	39 (51.3%)	<0.001
Mosaic attenuation on CT, n (%)	5 (5.2%)	24 (31.6%)	<0.001
Pleural involvement on CT, n (%)	3 (3.1%)	11 (14.5%)	0.01
CT small airway involvement score, n (%)			<0.001
Score 0	84 (87.5%)	25 (32.9%)	
Score 1	11 (11.5%)	39 (51.3%)	
Score 2	1 (1.0%)	12 (15.8%)	
Laboratory tests			
LDH, U/L	256.00 (231.50, 301.25)	270.50 (246.00, 313.25)	0.098
ESR, mm/h	24.00 (14.00, 34.00)	25.00 (13.00, 34.00)	0.738
Immune activation/inflammation phenotype			
sIL-2R, U/mL	176.85 (110.85, 284.70)	233.30 (134.78, 432.35)	0.027
CD14^+^HLA-DR^+^	96.94 (94.90, 97.95)	96.84 (93.71, 98.44)	0.868
Innate immune cell profile			
NK cells, cells/µL	112.78 (71.65, 188.08)	108.59 (65.03, 189.02)	0.555
Mo2, %	1.67 (1.02, 3.16)	1.63 (0.84, 2.78)	0.761
Adaptive lymphocyte profile			
CD4^+^ T, cells/µL	651.58 (396.91, 841.59)	715.71 (450.70, 1107.93)	0.122
CD8^+^ T, cells/µL	442.83 (302.55, 670.60)	593.14 (311.80, 785.74)	0.057
CD4/CD8, %	1.31 (1.09, 1.68)	1.27 (1.01, 1.59)	0.318
Treg/CD4, %	10.12 (8.40, 11.80)	9.19 (7.77, 11.07)	0.166
Cytokine			
IL-6, pg/mL	7.25 (3.00, 16.55)	7.65 (3.27, 21.73)	0.317
IL-8, pg/mL	26.70 (17.43, 55.58)	34.40 (14.40, 81.33)	0.388
IL-10, pg/mL	4.20 (3.40, 7.05)	5.35 (3.82, 8.85)	0.094
IFN-α, pg/mL	5.90 (5.50, 7.32)	5.80 (5.27, 7.50)	0.683
IFN-γ, pg/mL	5.90 (5.00, 16.10)	7.15 (5.00, 13.85)	0.346
IL-18, pg/mL	4.00 (4.00, 5.65)	4.00 (4.00, 6.80)	0.137
IL-17, pg/mL	10.05 (8.70, 11.53)	10.45 (9.35, 13.25)	0.092

Abbreviations: SAD, small airway dysfunction; CT, computed tomography; sIL-2R, soluble in-terleukin-2 receptor; CRP, C-reactive protein; LDH, lactate dehydrogenase; ESR, erythrocyte sedimentation rate; NK, natural killer; Treg, regulatory T cells. Continuous variables are presented as median (interquartile range, IQR), and categorical variables as n (%). Between-group comparisons were performed using the Wilcoxon rank-sum test for continuous variables and Fisher’s exact test for categorical variables. For immune markers displayed in the immune landscape analysis, between-group differences were calculated using log1p-transformed values, defined as log(x + 1). *p*-values are two-sided. Bronchoalveolar lavage was performed selectively for clinical indications and was not a protocol-mandated procedure.

**Table 2:** Multivariable logistic regression models for predicting small airway dysfunction in hospitalized children with *Mycoplasma pneumoniae* pneumonia.

Predictor	Unit	Reference/Comparison	BASE OR (95% CI)	*p*-Value	EXT OR (95% CI)	*p*-Value
Age	years	per 1-year increase	0.846 (0.677–1.058)	0.142	0.867 (0.694–1.083)	0.208
Sex	—	Male vs. Female	1.550 (0.653–3.680)	0.318	1.553 (0.649–3.715)	0.320
Wheezing	—	Yes vs. No	3.696 (1.254–10.895)	0.018	3.368 (1.139–9.961)	0.028
Moist rales	—	Yes vs. No	2.556 (0.914–7.152)	0.074	2.760 (0.962–7.919)	0.059
Wheezing in the past year	—	Yes vs. No	1.444 (0.522–3.997)	0.476	1.612 (0.571–4.548)	0.365
History of eczema	—	Yes vs. No	1.785 (0.763–4.177)	0.180	1.848 (0.782–4.365)	0.160
Bronchial wall thickening on CT	—	Yes vs. No	1.859 (0.394–8.764)	0.431	1.804 (0.385–8.451)	0.452
CT small airway involvement score	points	per 1-point increase	3.290 (0.770–14.054)	0.107	3.552 (0.826–15.272)	0.088
Chest CT total score	points	per 1-point increase	1.249 (0.858–1.819)	0.243	1.226 (0.838–1.795)	0.292
IL-17 (log1p)	pg/mL	per 1-unit increase in ln(IL-17 + 1)	—	—	2.466 (0.795–7.650)	0.117

Abbreviations: OR, odds ratio; CI, confidence interval; BASE, base model; EXT, extended model; CT, computed tomography; IL-17, interleukin-17. The BASE model included age, sex, wheezing, moist rales, wheezing within the previous year, history of eczema, bronchial wall thickening on CT, CT small airway involvement score, and chest CT total score. The EXT model included all variables in the BASE model plus log1p-transformed IL-17. Odds ratios and 95% confidence intervals were estimated from multivariable logistic regression models fitted in the multiply imputed dataset and pooled according to Rubin’s rules. All binary variables were coded as 0 = no and 1 = yes; sex was coded with female as the reference category (male = 1, female = 0). The CT small airway involvement score ranged from 0 to 2 points. IL-17 (log1p) was defined as ln(IL-17 + 1). *p*-values are two-sided.

## Data Availability

The data presented in this study are available on request from the corresponding author due to privacy.

## Supplementary Materials

The following supporting information can be downloaded at https://www.mdpi.com/article/10.3390/children13050713/s1: Figure S1: Distribution of bootstrap-corrected AUCs for the BASE and EXT models; Figure S2: Supplementary decision curve analysis and incremental net benefit of the EXT model compared with the BASE model; Table S1: Detailed baseline characteristics of hospitalized children with *Mycoplasma pneumoniae* pneumonia stratified by small air-way dysfunction status; Table S2: Variable-specific missingness rates in the candidate predictors; Table S3: Univariable logistic regression analyses of candidate predictors for small airway dysfunction.

## Abbreviations

The following abbreviations are used in this manuscript:

AUC	Area under the curve
ATS/ERS	American Thoracic Society/European Respiratory Society
BASE	Base model
BMP	Bronchial mucus plugs
BO	Bronchiolitis obliterans
CAP	Community-acquired pneumonia
CI	Confidence interval
CRP	C-reactive protein
CT	Computed tomography
DCA	Decision curve analysis
ESR	Erythrocyte sedimentation rate
EXT	Extended model
FEF25%	Forced expiratory flow at 25% of forced vital capacity
FEF50%	Forced expiratory flow at 50% of forced vital capacity
FEF75%	Forced expiratory flow at 75% of forced vital capacity
FEV1	Forced expiratory volume in 1 s
FVC	Forced vital capacity
IFN	Interferon
IL	Interleukin
IQR	Interquartile range
LASSO	Least absolute shrinkage and selection operator
LDH	Lactate dehydrogenase
MAE	Mean absolute error
MICE	Multiple imputation by chained equations
MMEF	Maximal mid-expiratory flow
MPP	*Mycoplasma pneumoniae* pneumonia
*M. pneumoniae*	*Mycoplasma pneumoniae*
NK	Natural killer
OR	Odds ratio
PA	Particle agglutination
PB	Plastic bronchitis
PCR	Polymerase chain reaction
PIBO	Post-infectious bronchiolitis obliterans
ROC	Receiver operating characteristic
RMPP	Refractory Mycoplasma pneumoniae pneumonia
SAD	Small airway dysfunction
sIL-2R	Soluble interleukin-2 receptor
SMPP	Severe Mycoplasma pneumoniae pneumonia
Treg	Regulatory T cells
Th	T-helper
