# Supplementary figures and images for: Early Risk Stratification for Subsequent Small Airway Dysfunction in Hospitalized Children with Mycoplasma Pneumoniae Pneumonia: A Retrospective Cohort Study
Source: Children (Basel). 2026 May 21;13(5):713. doi: 10.3390/children13050713 (PMC13204271; doi:10.3390/children13050713)

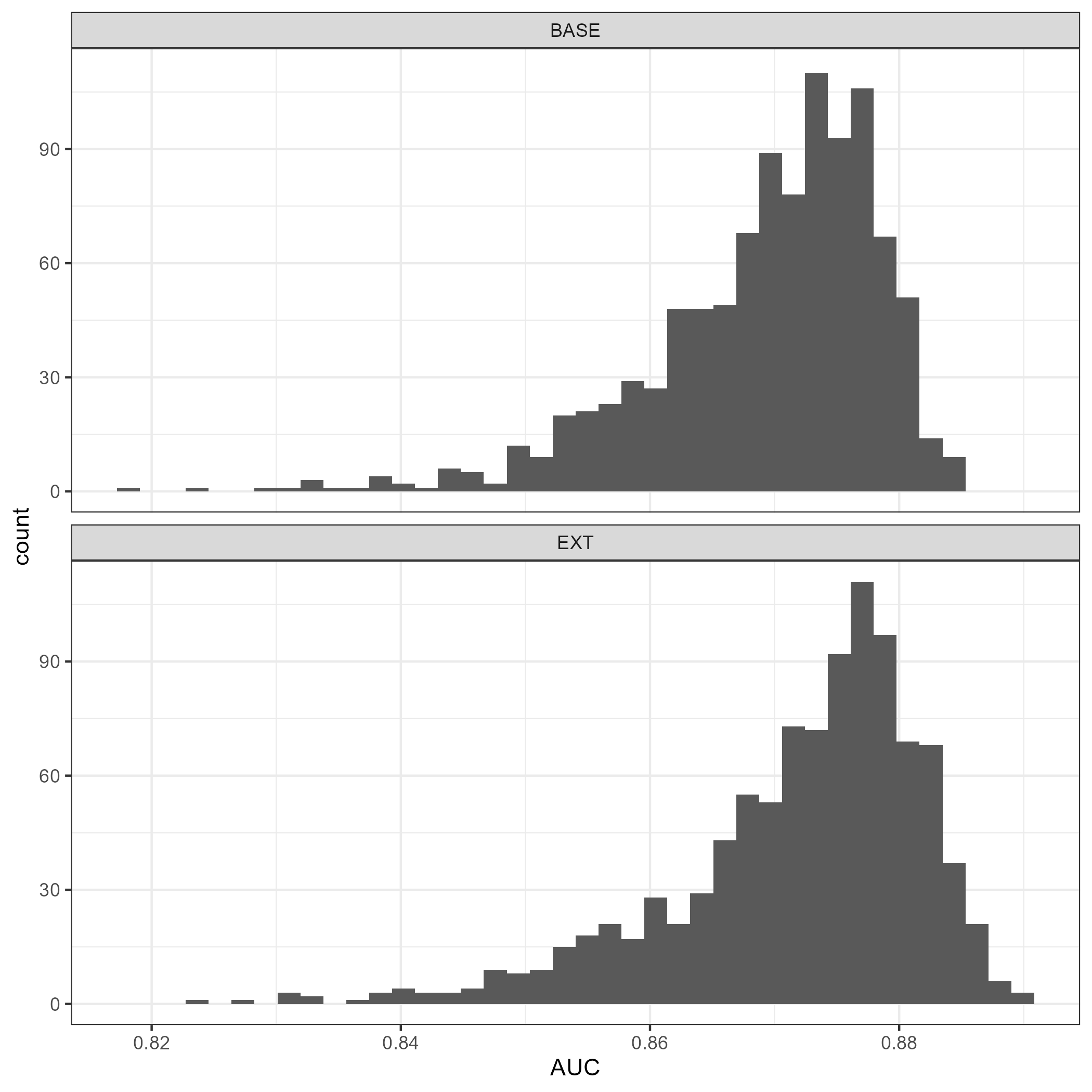

Supplement: Supplementary file 1 [file children-13-00713-s001.zip › Figure_S1_AUC_distribution.png]

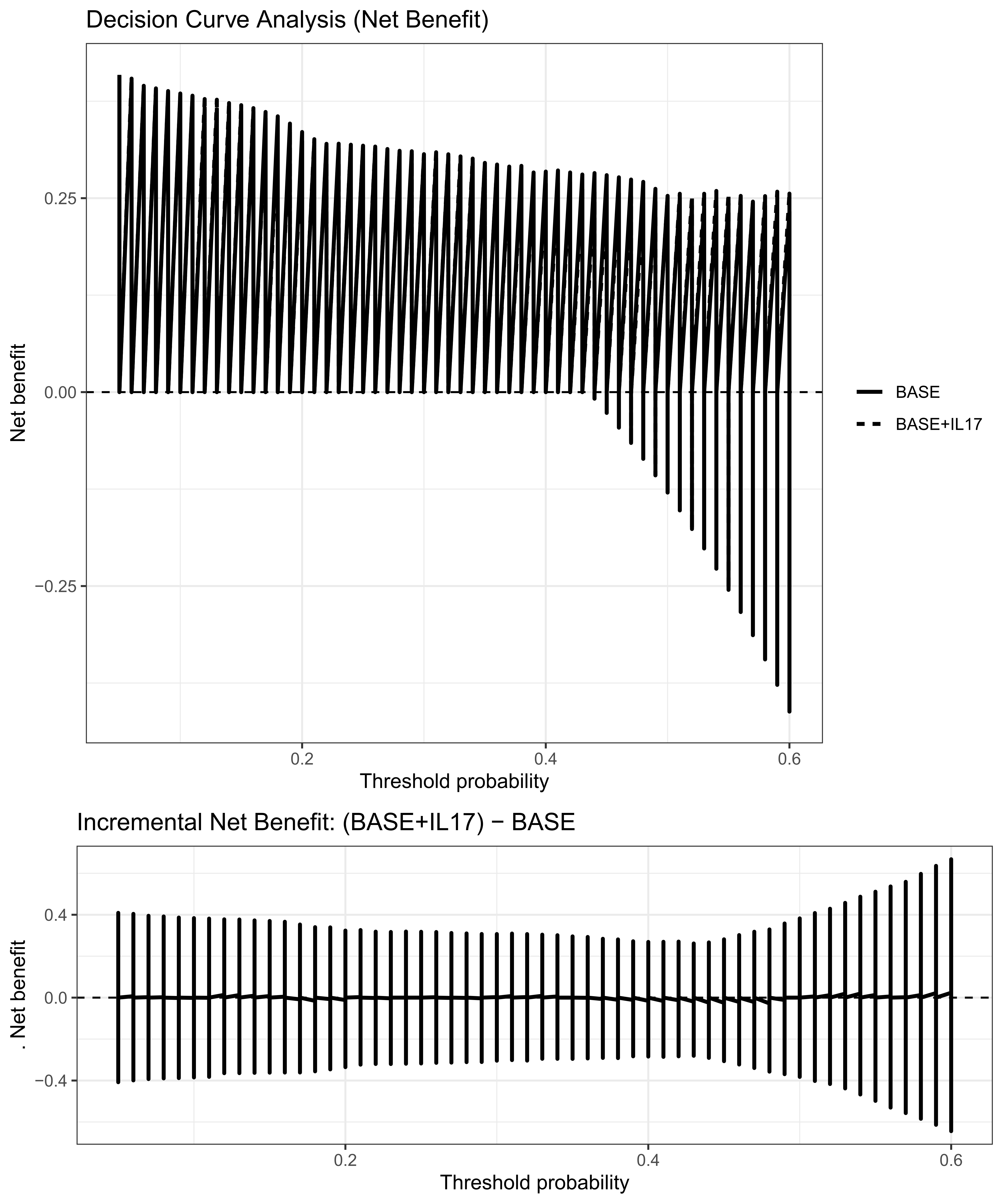

Supplement: Supplementary file 1 [file children-13-00713-s001.zip › Figure_S2_DCA_incremental_net_benefit.png]
